# Age at first sex in rural South Africa

**DOI:** 10.1136/sti.2008.033324

**Published:** 2009-03-13

**Authors:** N McGrath, M Nyirenda, V Hosegood, M-L Newell

**Affiliations:** 1 Infectious Disease Epidemiology Unit, London School of Hygiene and Tropical Medicine, London, UK; 2 Africa Centre for Health and Population Studies, University of KwaZulu Natal, South Africa; 3 Centre for Population Studies, London School of Hygiene and Tropical Medicine, London, UK; 4 Centre for Paediatric Epidemiology and Biostatistics, Institute of Child Health, UCL, London, UK

## Abstract

**Objectives:**

To identify factors associated with sexual debut and early age at first sex (AFS) among young men and women (12–25 years) in a population with a high prevalence and incidence of HIV in rural South Africa.

**Methods:**

Longitudinal data from four rounds (2003–7) of a prospective population-based HIV and sexual behaviour survey in rural KwaZulu-Natal were used to investigate the distribution and predictors of earlier first sex. Survival analyses were used, and each analysis considered men and women separately.

**Results:**

Among the 4724 women and 4029 men who were virgins at the beginning of the period, the median AFS was 18.5 and 19.2 years, respectively. In multivariable models, factors associated with earlier AFS across gender were periurban residence (vs rural), ever use of alcohol and knowing at least one person who had HIV, while school attendance had a significant protective effect. Other factors were important for one gender only. Maternal death was significantly associated with earlier AFS for women, in the same way that paternal death was for young men, while mother’s membership of the same household significantly delayed AFS of young men. The analysis of early first sex confirmed the same factors to be important as in the overall analyses for men and women.

**Conclusion:**

Given the association of individual, household and community level factors with sexual debut, a multisectorial approach to prevention and targeting in youth programmes is recommended.

South Africa has one of the highest HIV infection rates in the world,^1^ ^2^ and young people—particularly young women—continue to be at high risk.^3^ ^4^ Age at first sex (AFS) has been associated with increased risk of unplanned pregnancy and sexually transmitted infections, including HIV and human papillomavirus (HPV).^5^ Studies have examined early sexual activity largely as a potential risk factor for adverse outcomes rather than identifying the correlates of the timing of sexual debut per se.^3^ ^6^

Trends and differentials of AFS in sub-Saharan Africa have been explored,^7^ ^8^ as have certain determinants of AFS,^8^^–^10 primarily education^11^ and orphanhood.^12^ ^13^ Studies have estimated AFS in South Africa in various ways, typically using cross-sectional data from a single survey.^3^ ^4^ Few have used survival analysis,^14^ the most appropriate method for estimating the distribution of AFS from censored observations.^7^ Our study used longitudinal population-based data to identify factors associated with AFS in young men and women (12–25 years) and to ensure temporality of the observed associations in a population with a high prevalence and incidence of HIV in rural South Africa.

## METHODS

### Study population

The data for this study were obtained as part of a prospective population-based HIV and sexual behaviour survey in the rural Umkhanyakude district of KwaZulu-Natal, South Africa. Since 2000, the Africa Centre Demographic Information System (ACDIS) has collected longitudinal social, demographic and health data^15^ in a Zulu speaking population of approximately 86 000 (see www.africacentre.ac.za). Individuals who move or belong to more than one household are tracked at each household. Therefore, at any one time, individuals can be resident at one household while being a member of multiple households.^16^ ^17^ Sexual behaviour questionnaires were administered annually to all male residents aged 15–54 years and female residents aged 15–49 years in 2003/4 to 2007. The 2003/4 survey included additional questions about knowledge and awareness of HIV. Details about the data collection methods have been published previously.^15^

The age range 12–25 years was chosen because these individuals were eligible to participate in at least one sexual behaviour survey during the period and a review of Kaplan-Meier estimates of survival until first sex indicated that the hazard was close to zero beyond the age of 25 years for women.

### Sample

The period of observation for this analysis was from 1 January 2003 to 31 December 2007. Individuals aged 12–25 years and resident in the surveillance area on 1 January 2003 who reported never having had sex by the start of the period were considered at risk of first sex. These criteria meant that the analytical sample included 4724 women and 4029 men (table 1).

**Table 1:** Characteristics of study population

		Men	Women
Number resident in ACDSA on 1 January 2003 aged 12–25 years		10089	10469
Number who participated in at least one sexual behaviour survey during 2003–7 (% of number resident)		6728 (67%)	7917 (76%)
Ever had sex			
Yes		3625	5038
No		2849	2664
Missing		254	215
Number whose sexual debut was before the start of the observation period (total, %)*		2345 (6374; 37%)	2924 (7648; 38%)
Median (IQR) age (years) of those who had already sexually debuted		19.7 (18.0–21.8)	20.8 (19.1–22.8)
Analysis population: number who had not already sexually debuted by the start of the observation period		4029	4724

ACDSA, Africa Centre Demographic Surveillance Area; IQR, interquartile range.

*AFS was missing for 48 women (1%) and 95 men (3%); 6 other women and 5 men were excluded because their AFS report was higher than their age at time of interview.

### Measures

In each sexual behaviour survey, women were asked if they had ever had sex and at what age they first had sex. Men were asked both questions in the 2003/4 survey but from 2005 onwards were only asked at what age they first had sex. Table 2 shows the consistency of AFS reporting among those who sexually debuted during the observation period. Factors explored as potential determinants of AFS included (1) individual-level variables: religious affiliation, ever use of alcohol, smoking, school attendance and grade-for-age; (2) household-level variables: household size, parental membership of the same household, parental death before sexual debut, household assets and place of residence (urban, periurban and rural); (3) knowledge and awareness of HIV, ever use of alcohol and self-reported general health status were available for those who participated in the 2003/4 survey round (56% of women and 40% of men). HIV knowledge and awareness included questions about HIV transmission, whether they knew people with HIV, their perceptions of whether a healthy-looking person could have HIV, whether a lot of people in the community were in danger of becoming HIV-infected and whether neighbours would give help and acceptance if the respondent became ill with AIDS. Ever and current smoking reported in 2003 were examined for men only since very few women reported this behaviour.

**Table 2:** Consistency of age at first sex (AFS) reporting among those whose sexual debut was during the observation period

	Men(N = 1176)	Women(N = 2051)
Only one AFS	813 (69%)	986 (48%)
AFS considered the mid point between two survey rounds when survey reports transitioning from never having had sex to having had sex	–	85 (4%)
⩾2 reports: consistent	89 (8%)	290 (14%)
⩾2 reports: within a year	105 (9%)	226 (11%)
⩾2 reports: inconsistent but most likely AFS possible to identify	52 (4%)	232 (11%)
⩾2 reports: inconsistent, mean AFS used	117 (10%)	232 (11%)

Parental membership of the household, parental survival status, education grade-for-age, school attendance and assets were included in the analyses as time-varying covariates, benefiting from the updated information collected during the period.

### Statistical analysis

Analyses were conducted to determine factors significantly associated with sexual debut and to explore factors associated with early AFS (defined as before the age of 17 years, the 25th percentile of AFS for men and women in the overall analyses). Each analysis considered men and women separately because we expected the factors associated with first sex to differ by gender. Survival analysis methods for the overall analyses were used to accommodate censoring of those who had not yet had sex by the time of their last sexual behaviour survey or their 25th birthday during the period.^7^ For analyses of early sex, the sample was restricted to virgins who were <17 years of age at the start of the observation period (censoring at the end of the period or their 17th birthday, whichever came first). The median AFS and interquartile range (IQR) were calculated by different characteristics. Mantel-Cox hazard ratios were used for univariate analyses.^18^ The results are the hazard of first sex—that is, the chance that an individual has experienced first sex at age n, given that they had not had sex before their nth birthday. The equality of the survival functions across levels of each factor was examined using a log-rank test (Mantel-Cox χ^2^ test). Variables significant in univariate analyses, as well as other variables previously indicated in the literature as important, were considered in multivariable Cox proportional hazard models using the Breslow method for ties.^19^ Statistical significance was set at an alpha level of 0.05. For both the univariate and multivariate models, hazard ratios >1 indicate that an individual in the category is more likely to sexually debut than an individual in the referent category (ie, the AFS is younger than the AFS for the reference category).

The assumption of proportional hazards for each factor was tested by examining the scaled Schoenfeld residuals for a trend with age.^20^ In women, school attendance violated the proportional hazards assumption. Therefore, the univariate analysis was conducted in three separate age groups (<18 years, 18–20 years and 21+ years) and school attendance was not included in the overall multivariable analyses for women. However, it was included in the analysis of early sex among women since the proportional hazards assumption was appropriate for school attendance in this subpopulation.

Analyses were conducted using Stata Version 10.0 (StataCorp, Texas, USA).

## RESULTS

### Women

Among the 4724 women who were virgins at the beginning of the period, 2051 (43%) reported ever having sex during 11 732 person-years of follow-up. The median AFS was 18.5 years (IQR 17.1–20.3). Table 3 shows the crude and adjusted hazard ratios for AFS among women for selected explanatory variables. Univariate results suggest that the hazard of first sex was statistically significantly higher for women whose mother or father had died, were of Zionist or no religion compared with the Shembe religion, and had ever used alcohol. The hazard of first sex was statistically significantly lower for women living in a rural area compared with a periurban setting, whose mother or father was a co-member of the same household, who did not know any relative with HIV, thought the community was not in danger from HIV and were attending school (among women aged <21 years). Religion, place of residence, mother’s survival status, ever use of alcohol and the number of relatives known to have HIV were retained in the final multivariable model, with the same direction of association seen univariately. The indicators for missing data about mother’s survival and HIV knowledge were also significant in the multivariable model. The multivariable model conformed to the proportional hazards assumption (global χ^2^ 9.17, p = 0.69, 12 df).

**Table 3:** Percentage distribution, median age at first sex (AFS) and crude and adjusted hazard ratios for first sex in women

Variable	N (% of population)†	Median (IQR) AFS	Crude HR (95% CI)	p Value*	Adjusted HR (95% CI)‡	p Value**
Religion				0.004		0.01
Shembe	884 (19)	18.8 (17.3–20.6)	1.0		1.0	
Zionist	1556 (33)	18.3 (17.0–20.0)	1.20 (1.06 to 1.36)		1.18 (1.05 to 1.34)	
West Christian	1371 (29)	18.5 (17.1–20.3)	1.07 (0.94 to 1.22)		1.03 (0.91 to 1.18)	
None	117 (2)	18.0 (16.8–19.6)	1.41 (1.07 to 1.86)		1.36 (1.03 to 1.80)	
Other	331 (7)	18.9 (17.9–20.5)	0.96 (0.79 to 1.18)		0.95 (0.77 to 1.16)	
Geographical location				0.007		0.05
Rural	3297 (70)	18.7 (17.2–20.4)	1.0		1.0	
Urban	196 (4)	18.5 (17.0–19.9)	1.11 (0.86 to 1.43)		1.13 (0.87 to 1.46)	
Periurban	1231 (26)	18.2 (17.0–19.9)	1.17 (1.06 to 1.29)		1.13 (1.02 to 1.25)	
Education (grade-for-age)				0.2		
Age appropriate	3665 (31)	18.7 (17.2–N/A)	1.0			
Behind by 1 year	2809 (24)	18.5 (17.0–20.4)	1.02 (0.88 to 1.18)			
Behind 2+ years	3642 (31)	18.5 (17.1–20.3)	1.05 (0.91 to 1.20)			
Complete secondary	1513 (13)	18.4 (17.0–20.0)	1.04 (0.85 to 1.27)			
School attendance <18 years				<0.001		
Attending school	7271 (81)	(17.3–N/A)	1.0			
Not in school	298 (3)	16.4 (16.0–17.6)	3.86 (3.24 to 4.59)			
Complete secondary	904 (10)	(17.0–N/A)	1.17 (0.93 to 1.46)			
School attendance 18–21 years				<0.001		
Attending school	1534 (67)	20.3 (19.0–N/A)	1.0			
Not in school	203 (9)	18.9 (18.4–19.7)	2.48 (2.07 to 3.00)			
Complete secondary	415 (18)	19.8 (18.8–N/A)	1.34 (1.12 to 1.60)			
School attendance 21+ years				0.004		
Attending school	127 (29)	23.4 (22.1–N/A)	1.0			
Not in school	91 (21)	22.7 (21.8–24.7)	1.53 (0.92 to 2.53)			
Complete secondary	194 (45)	22.1 (21.7–24.0)	1.98 (1.31 to 3.0)			
Mother member of household				0.015		
No	3947 (34)	18.3 (17.0–20.0)	1.0			
Yes	7784 (66)	18.6 (17.2–20.4)	0.89 (0.81 to 0.98)			
Father member of household				0.01		
No	6714 (34)	18.4 (17.0–20.0)	1.0			
Yes	5018 (66)	18.7 (17.2–20.5)	0.89 (0.81 to 0.97)			
Mother dead				<0.001		<0.001
No	9744 (83)	18.6 (17.1–20.4)	1.0		1.0	
Yes	1645 (14)	18.1 (17.0–19.7)	1.23 (1.10 to 1.39)		1.23 (1.09 to 1.38)	
Missing	342 (3)	18.0 (16.7–19.4)	1.53 (1.22 to 1.92)		1.45 (1.15 to 1.82)	
Father dead						
No	8238 (70)	18.6 (17.2–20.4)	1.0	0.06		
Yes	2926 (25)	18.3 (17.0–20.0)	1.10 (1.00 to 1.21)			
Ever taken alcohol				0.002		0.02
No	2547 (54)	18.7 (17.2–20.4)	1.0		1.0	
Yes	111 (2)	17.4 (16.6–19.1)	1.50 (1.17 to 1.92)		1.42 (1.10 to 1.82)	
Missed 2003 survey	2066 (44)	18.4 (17.0–20.0)	1.14 (1.04 to 1.25)		1.13 (1.03 to 1.25)	
Community in danger				0.04		
No/don’t know	1262 (27)	18.8 (17.2–20.5)	1.0			
Yes	1401 (30)	18.4 (17.1–20.3)	1.12 (1.00 to 1.26)			
Know relatives who have/had AIDS§				0.006		0.03
No	2457 (52)	18.7 (17.2–20.5)	1.0		1.0	
Yes (1+)	198 (4)	18.0 (16.6–19.4)	1.32 (1.08 to 1.60)		1.26 (1.03 to 1.53)	
HIV is transmitted by sharing utensils¶				0.89		
No	1874 (40)	18.6 (17.2–20.4)	1.0			
Yes/don’t know	874 (17)	18.5 (17.0–20.4)	0.99 (0.87 to 1.12)			

HR, hazard ratio; N/A, not applicable.

*Univariate likelihood ratio test p value.

†Totals vary because of missing values. The percentage distribution is based on the population of 4724 virgins. For time-varying characteristics it is based on person-years and one individual could contribute to different categories during the study period.

‡N = 4724. Missing indicators were used to represent the individuals with no information in multivariable models, adjusted for all other factors in the column, factors with a likelihood ratio test p value ⩽0.05. School attendance was not considered for multivariable analyses.

§The variable “knowing people with HIV” violated the proportional hazards assumption univariately. Knowing relatives with HIV was highly correlated with knowing people and neighbours with HIV, and only the variable “knowing relatives with HIV” was considered for the multivariable analysis.

¶Similar non-significant results were attained for the questions about other modes of HIV transmission.

**Likelihood ratio test p value for the contribution of the variable to the multivariable model.

Note: Hazard ratios >1 indicate that the AFS is younger than the AFS for the reference category.

In the analysis of early sex, 3406 women were <17 years of age at the start of the period. During 7320 person-years of follow-up, 579 reported sexual debut. Knowing *anyone* with HIV rather than relatives was considered in this multivariable analysis because it did not violate the proportional hazards assumption. School attendance was strongly associated with AFS in the multivariable model, with non-attendance associated with more than a four times greater hazard of first sex, holding age constant (table 4). Religion, mother’s survival status and knowing someone with HIV were also statistically significant. Ever use of alcohol was no longer statistically significant, but had a similar estimate of association to women’s AFS as seen in the overall analysis.

**Table 4:** Multivariable model for early first sex in young women

Variable	Adjusted HR (95% CI)(model A)	p Value*
Religion		0.05
Shembe	1.0	
Zionist	1.19 (0.94 to 1.51)	
West Christian	0.96 (0.75 to 1.25)	
None	1.26 (0.79 to 2.03)	
Other	0.68 (0.44 to 1.05)	
School attendance		<0.001
Attending school	1.0	
Not in school	4.19 (3.27 to 5.38)	
Mother dead		0.03
No	1.0	
Yes	1.17 (0.94 to 1.46)	
Missing	1.66 (1.13 to 2.42)	
Ever taken alcohol		0.17
No	1.0	
Yes	1.46 (0.87 to 2.45)	
Know someone who has/had AIDS		0.02
No	1.0	
Yes (1+)	1.45 (1.07 to 1.96)	
Missed 2003 survey	1.21 (1.00 to 1.47)	
Geographical location		0.54
Rural	1.0	
Urban	1.26 (0.80 to 1.98)	
Periurban	1.06 (0.88 to 1.28)	

*Likelihood ratio test p value for the contribution of the variable to the multivariable model.

### Men

Among the 4029 men who were virgins at the beginning of the period, 1176 (29%) reported ever having sex during 10 598 person-years of follow-up. The median (IQR) AFS was 19.2 (17.0–24.8) years. Table 5 shows the crude and adjusted hazard ratios for AFS in men for selected explanatory variables. Univariate results suggest that the hazard of first sex was statistically significantly higher for men who were not attending school, whose mother or father had died, for those who had ever used alcohol or tobacco, and those who knew that a healthy-looking person could have HIV. The hazard of having experienced first sex was statistically significantly lower for those who were one or more years behind in school for their age, living in a rural area compared with a periurban setting, whose mother or father was a co-member of the same household, who did not know anyone with HIV, thought the community was not in danger from HIV and that HIV could be transmitted by sharing utensils. Father’s survival status, mother’s membership of the same household, ever alcohol use, ever smoked, school attendance and grade-for-age, geographical location, whether a healthy-looking person could have HIV and the number of people known to have HIV were retained as significant factors in the final multivariable model, with the same direction of association seen univariately. The indicator representing those who had no HIV knowledge data (no 2003 survey) was also significant in the multivariable model. The multivariable model conformed to the proportional hazards assumption (global χ^2^ 15.48, p = 0.42, 15 df). The change in the hazard ratio estimates between the univariate and multivariate results (for school attendance, ever use of alcohol, ever smoking and knowledge that a healthy-looking person can have HIV) suggest there might be confounding. We conducted additional analyses to explore this and found that, despite a low prevalence of these behaviours overall, ever use of alcohol and ever smoking were correlated (ρ = 0.54). Their prevalence varied significantly depending on school attendance status, with the highest prevalence of alcohol and smoking among the “not in school” category. Interaction terms were explored but none were statistically significant.

**Table 5:** Percentage distribution, median age at first sex (AFS) and crude and adjusted hazard ratios for first sex in men

Variable	N (% of population)†	Median (IQR) AFS	Crude HR (95% CI)	p Value*	Adjusted HR (95% CI)‡	p Value**
Geographical location				0.008		0.04
Rural	2886 (72)	19.4 (17.2–N/A)	1.0		1.0	
Urban	97 (2)	20.1 (16.9–N/A)	0.96 (0.64 to 1.43)		0.85 (0.57 to 1.26)	
Periurban	1046 (26)	18.6 (16.6–22.9)	1.22 (1.07 to 1.38)		1.16 (1.03 to 1.32)	
Education (grade-for-age)				<0.001		<0.001
Age appropriate	2287 (22)	18.5 (16.6–N/A)	1.0		1.0	
Behind by 1 year	2249 (21)	19.6 (17.1–N/A)	0.72 (0.59 to 0.87)		0.73 (0.60 to 0.87)	
Behind 2+ years	5226 (49)	19.4 (17.2–N/A)	0.71 (0.61 to 0.84)		0.71 (0.61 to 0.84)	
Complete secondary	773 (7)	18.5 (16.9–22.1)	0.88 (0.67 to 1.16)			
School attendance				<0.001		<0.001
Attending school	8564 (80)	19.5 (17.0–N/A)	1.0		1.0	
Not in school	599 (6)	18.5 (16.8–21.0)	1.55 (1.27 to 1.90)		1.44 (1.17 to 1.77)	
Complete secondary	773 (7)	18.5 (16.9–22.1)	1.22 (0.98 to 1.51)		0.90 (0.71 to 1.15)	
Mother member of household				0.003		0.003
No	3982 (38)	18.9 (16.8–22.1)	1.0		1.0	
Yes	6616 (62)	19.6 (17.2–N/A)	0.84 (0.75 to 0.94)		0.83 (0.74 to 0.94)	
Father member of household				0.01		
No	6211 (59)	19.0 (17.0–22.6)	1.0			
Yes	4387 (41)	19.8 (17.2–N/A)	0.86 (0.76 to 0.97)			
Mother dead				0.03		
No	8607 (81)	19.3 (17.1–N/A)	1.0			
Yes	1594 (15)	18.7 (16.6–22.1)	1.19 (1.02 to 1.38)			
Father dead				0.001		0.002
No	7282 (69)	19.5 (17.1–N/A)	1.0		1.0	
Yes	2816 (27)	18.7 (16.8–21.9)	1.23 (1.09 to 1.39)		1.21 (1.07 to 1.37)	
Ever taken alcohol				<0.001		<0.001
No	1286 (32)	19.8 (17.4–N/A)	1.0		1.0	
Yes	330 (8)	16.3 (14.5–18.6)	1.89 (1.55 to 2.30)		1.60 (1.30 to 1.98)	
Missed 2003 survey	2413 (60)	19.2 (17.1–N/A)	1.02 (0.89 to 1.17)		1.46 (1.15 to 1.86)	
Ever smoked				<0.001		<0.001
No	1551 (38)	19.3 (16.9–N/A)	1.0		1.0	
Yes	84 (2)	16.9 (16.3–18.3)	2.70 (1.99 to 3.66)		1.99 (1.45 to 2.73)	
Community in danger				0.02		
No/don’t know	748 (19)	19.3 (16.8–N/A)	1.0			
Yes	846 (21)	18.9 (16.8–22.8)	1.23 (1.03 to 1.48)			
Know people who have/had AIDS§				<0.001		<0.001
No	1341 (33)	19.4 (17.0–N/A)	1.0		1.0	
Yes (1+)	274 (7)	17.8 (16.4–20.4)	1.74 (1.41 to 2.14)		1.67 (1.36 to 2.06)	
HIV is transmitted by utensils¶				0.01		
No	1027 (25)	18.8 (16.6–22.6)	1.0			
Yes/don’t know	588 (15)	20.3 (17.8–N/A)	0.77 (0.61 to 0.97)			
A healthy-looking person can have HIV				<0.001		<0.001
No	374 (9)	20.6 (17.7–N/A)	1.0		1.0	
Yes	1242 (31)	18.6 (16.5–21.9)	1.63 (1.29 to 2.06)		1.48 (1.16 to 1.87)	

HR, hazard ratio; N/A, not applicable.

*Univariate likelihood ratio test p value.

†Totals vary because of missing values. The percentage distribution is based on the population of 4029 virgins. For time-varying characteristics it is based on person-years and one individual could contribute to different categories during the study period.

‡N = 4029. Missing indicators were used to represent the individuals with no information in multivariable models, adjusted for all other factors in the column, factors with a likelihood ratio test p value ⩽0.05.

§Knowing people with HIV was highly correlated with knowing relatives and neighbours with HIV and only the variable “knowing people with HIV” was considered for the multivariable analysis.

¶Similar non-significant results were attained for the questions about other modes of HIV transmission.

**Likelihood ratio test p value for the contribution of the variable to the multivariable model.

Note: Hazard ratios >1 indicate that the AFS is younger than the AFS for the reference category.

In the analysis of early sex, 3103 men were <17 years of age at the start of the period. During 6869 person-years of follow-up, 553 reported sexual debut. Ever use of alcohol, grade for age, place of residence, mother being a member of the household, knowing at least one person who had HIV and knowing that HIV cannot be transmitted through sharing utensils remained significant in a multivariable model (data not shown), with similar associations to the AFS for men as those seen in the overall analysis.

## DISCUSSION

The median AFS for young women and men was 18.5 years and 19.2 years, respectively. This is consistent with Bakilana’s estimate of 18 years for South African women using 1998 DHS data and a survival analysis approach.^14^ Bakilana found that South African women tended to enter into sexual relations later than Tanzanians and more or less at the same age as Zimbabweans. Similarly, Hallett *et al* found that the median AFS for women was 18 years and for men was 19 years in rural Zimbabwe in 2000.^9^ Different methods for summarising AFS will give slightly different estimates.^21^ Our study design selected only virgins aged 12–25 years at the beginning of the study period. By excluding those who debuted early, we may therefore overestimate the median AFS. In contrast, median AFS estimates in a 2003 South African nationally representative survey by Pettifor *et al*^3^ (16 years for men and 17 years for women) may have been underestimated, given that AFS was estimated among 15–24-year-olds who had ever had sex at the time of the cross-sectional survey. Our estimate is, however, similar to the median AFS of 18 years for both sexes reported from the Nelson Mandela HSRC national survey (2002), based on a larger age group of 15–49-year-olds.^4^ The sex differential in AFS between men and women in our study is also consistent with other South African data showing that men had partners aged, on average, 1 year younger than themselves, while women had partners who were on average 4 years older.^3^

Time-to-event (Cox) analyses were used because it allows the inclusion of individuals who did not sexually debut during the observation period, thereby comparing the number of individuals at risk (virgins) in each group at multiple points in time rather than excluding those who remain virgins (a biased analysis). However, the hazard ratios estimated in these Cox models do not translate directly into information about the actual age at first sex, unless the data are fit to an underlying parametric survival distribution.

The longitudinal data available in ACDIS provide an opportunity to examine AFS and its determinants, ensuring temporality of the determinants relative to first sex as well as permitting the updating of these factors during the time that an individual remained at risk of sexual debut. We found several factors significantly associated with AFS in men and women, with the majority being important for one sex but not the other.

School attendance was significantly associated with later sexual debut among both men and women. The fact that school attendance needed to be considered separately for women aged <18 years, 18–21 years and 21+ years in order to fulfil the proportional hazards assumption suggests that the protective effect of school is not constant in women and, in part, depends on age. Other studies also report a strong association between school attendance and later AFS in young women.^11^ Like us, other authors note the difficulty in disentangling the temporality of pregnancy and school non-attendance between rounds of data collection. We found no association of AFS with grade-for-age among women. In contrast, both school attendance and being behind in terms of grade-for-age were significantly associated with a later AFS for young men. We are unaware of previous studies examining this association in young men. A review by Hargreaves and Boler found no “striking gender differences” in terms of the impact of education on HIV vulnerability which included early AFS.^11^ Our results are consistent with other literature that suggests the school environment is associated with later first sex in men and women. The additional suggestion that being behind in school has a protective effect for men warrants further investigation. Notably, 49% of the men were ⩾2 years behind their grade-for-age compared with 31% of the women in these analyses.

Many studies have focused on orphanhood and its impact on sexual behaviour outcomes,^12^ ^13^ ^22^ ^23^ education and household socioeconomic status.^24^ ^25^ We found maternal death was significantly associated with earlier AFS for women, in the same way that paternal death was for young men. The ACDIS data enable us to explore whether parental comembership of a young person’s household is associated with AFS. Mother’s membership of the same household significantly delayed AFS of young men, after adjusting for father’s survival status and other factors. This positive influence on AFS is consistent with observations in Côte d’Ivoire^26^ and the USA.^27^ In the study population, young people were much more likely to be a member of the same household as their mother than their father.^28^

**Take-home messages**

- For both sexes, school attendance was significantly associated with later age at first sex (AFS).
- For both sexes, periurban residence (versus rural), ever use of alcohol and knowing someone who had HIV were associated with earlier AFS.
- Other factors significantly associated with AFS were important for one gender only, including maternal death for women and paternal death for men.
- Given the influence of individual, household and community-level factors, a multisectorial approach to prevention and targeting in youth programmes is recommended.

Religious affiliation was significantly associated with AFS in women but not in men. Religion was also shown to be a predictor of AFS in a study in Nigeria.^10^ Whether this reflects varying levels of religiosity between different churches, the type of messages about sex and institutional pressure within church or peer norms within a church-based social network in delaying first sex is unclear.^27^ In many African populations, first marriage is an important determinant of AFS, particularly for women. However, in our analysis, so few young men and women were married by the age of 25 years that it was not possible to explore marriage as a determinant of AFS. Data from ACDIS in 2006 indicate that only 7% of 25–29-year-old women had ever married, while only 1% of men of the same age group had ever married.^29^

For both sexes, AFS was significantly associated with place of residence, ever use of alcohol and knowing at least one person who had HIV. The association of alcohol use with earlier AFS has been observed in some non-African populations.^27^ ^30^ Access to alcohol may also reflect the social environment in which these young people live, one that may have facilitated earlier sexual debut by introducing possible sexual partners and opportunities. The data do not specify whether alcohol was used at the time of first sex.^31^ Although ever having smoked was a rare event (5% in those with non-missing data), it was correlated with alcohol use and was an independent risk factor of earlier AFS among men, even after adjustment for ever use of alcohol and other factors. Smoking and alcohol use may identify young “risk takers”, people who are also more likely to explore sexual activity early. Furthermore, living in a more rural rather than a periurban area was associated with later AFS in men and women. The periurban area is densely, often informally, settled. These findings suggest that there may be a constellation of community-level factors that influence the timing of first sex.

Knowing at least one person with HIV was associated with significantly earlier AFS. This may disappoint those hoping that first-hand experience of HIV might delay sexual debut. However, despite the extensive epidemic in this area,^1^ ^2^ the majority of young people reported knowing nobody in 2003/4 with HIV. This may suggest a lack of disclosure and denial, mirrored by the evenly split responses by both young men and women as to whether HIV is a danger to their community.

The analysis of early first sex (defined as before age 17 years) showed that the variables identified as important factors in the overall analysis for men and women were generally statistically significant in the subgroup analyses as well. This may not be surprising, given the assumption of proportional hazards over time (ie, age) underlying the overall analysis. However, the fact that these factors were significant in this less powered analysis may suggest that efforts to delay first sex can use the same prevention messages to target both those aged under 17 years and those under 25 years of age.

### Limitations of the study

Almost 25% of the men in these analyses had not sexually debuted by the age of 25 years. This could in part be an artifact of data collection since the male—but not female—surveys identified men who had “never had sex” from the question “How old were you when you started to have sex?” rather than directly asking about ever having sex. Generally, sexual behaviour surveys are subject to social desirability bias^32^ and inconsistent reporting of AFS for other reasons.^33^ We were able to cross-check internal consistency using questions on paternity and recent sexual partnerships.

In women, missing data on maternal survival was associated with earlier AFS in the multivariable models. However, these data were only missing for 3% of the person time at risk and may be a proxy for recent maternal death or lack of maternal involvement. For both sexes, missing HIV knowledge data generally reflect a younger age in 2003 since the lower limit for participation was 15 years in all surveys. However, the significant association of these missing data with earlier AFS may also reflect unmeasured confounding in selection into later survey rounds or may be due to chance. In addition, our inability to update information about alcohol use, smoking and HIV knowledge during the period means that, for some individuals, the data may not reflect knowledge and behaviour at the time of sexual debut.

Studies have shown that the risk of HIV infection is lower among women who begin sexual activity later,^34^ ^35^ and that sex education and HIV education interventions can successfully delay sexual debut in developing countries.^36^ Our findings provide additional insights about the timing and factors associated with sexual debut in a population experiencing a severe HIV epidemic. The specific vulnerabilities of youth at the individual, household and community level call for a multisectorial approach to prevention programmes targeting youth to reduce HIV risk,^37^ ^38^ as well as the targeting of the timing of interventions (eg, while young people are still in school).

## References

[1] Welz T Hosegood V Jaffar S Continued very high prevalence of HIV infection in rural KwaZulu-Natal, South Africa: a population-based longitudinal study. AIDS 2007;21:1467–72 17589193 10.1097/QAD.0b013e3280ef6af2

[2] Barnighausen T Tanser F Mbizana C High HIV incidence despite high prevalence in rural South Africa: findings from a prospective population-based study. AIDS 2008;22:139–44 18090402 10.1097/QAD.0b013e3282f2ef43

[3] Pettifor AE Rees HV Kleinschmidt I Young people’s sexual health in South Africa: HIV prevalence and sexual behaviors from a nationally representative household survey. AIDS 2005;19:1525–34 16135907 10.1097/01.aids.0000183129.16830.06

[4] Shisana O Rehle T Simbayi L South African National HIV Prevalence, HIV Incidence, Behaviour and Communication Survey, 2005. Cape Town: HSRC Press, 2005

[5] Cooper D Hoffman M Carrara H Determinants of sexual activity and its relation to cervical cancer risk among South African Women. BMC Public Health 2007;7 10.1186/1471-2458-7-341 PMC2228293 18042284

[6] Harrison A Cleland J Gouws E Early sexual debut among young men in rural South Africa: heightened vulnerability to sexual risk? Sex Transm Infect 2005;81:259–61 15923298 10.1136/sti.2004.011486 PMC1744981

[7] Żaba B Pisani E Slaymaker E Age at first sex: understanding recent trends in African demographic surveys. Sex Transm Infect 2004;80 (Suppl 2):ii28–35 15572637 10.1136/sti.2004.012674 PMC1765851

[8] Gupta N Mahy M Sexual initiation among adolescent girls and boys: trends and differentials in sub-Saharan Africa. Arch Sex Behav 2003;32:41–53 12597271 10.1023/a:1021841312539

[9] Hallett TB Lewis JJ Lopman BA Age at first sex and HIV infection in rural Zimbabwe. Stud Fam Plann 2007;38:1–10 17385378 10.1111/j.1728-4465.2007.00111.x

[10] Fatusi AO Blum RW Predictors of early sexual initiation among a nationally representative sample of Nigerian adolescents. BMC Public Health 2008;8 10.1186/1471-2458-8-136 PMC2390536 18439236

[11] Hargreaves J Boler T Girl power: the impact of girls’ education on HIV and sexual behaviour Johannesburg: ActionAid International, 2006

[12] Birdthistle IJ Floyd S Machingura A From affected to infected? Orphanhood and HIV risk among female adolescents in urban Zimbabwe. AIDS 2008;22:759–66 18356606 10.1097/QAD.0b013e3282f4cac7

[13] Gregson S Nyamukapa CA Garnett GP HIV infection and reproductive health in teenage women orphaned and made vulnerable by AIDS in Zimbabwe. AIDS Care 2005;17:785–94 16120495 10.1080/09540120500258029

[14] Bakilana A Age at sexual debut in South Africa. Afr J AIDS Res 2005;4:1–5 10.2989/16085900509490335 25865635

[15] Tanser F Hosegood V Barnighausen T Cohort Profile: Africa Centre Demographic Information System (ACDIS) and population-based HIV survey. Int J Epidemiol 2008;37:956–62 17998242 10.1093/ije/dym211 PMC2557060

[16] Hosegood V Timaeus IM Household composition and dynamics in KwaZulu-Natal, South Africa: mirroring social reality in longitudinal data collection. van der Walle E, ed. African households. Census data New York: M E Sharpe, 2005:58–77

[17] Hosegood V Benzler J Solarsh G Population mobility and household dynamics in rural South Africa: implications for demographic and health research. S Afr J Demogr 2005;10:43–67

[18] Clayton DG Hills M Statistical models in epidemiology Oxford: Oxford University Press, 1993

[19] Breslow NE Covariance analysis of censored survival data. Biometrics 1974;30:89–99 4813387

[20] Schoenfeld D Residuals for the proportional hazards regresssion model. Biometrika 1982;69:239–41

[21] Slaymaker E A critique of international indicators of sexual risk behaviour. Sex Transm Infect 2004;80:13–21 10.1136/sti.2004.011635 PMC1765852 15572635

[22] Thurman TR Brown L Richter L Sexual risk behavior among South African adolescents: is orphan status a factor? AIDS Behav 2006;10:627–35 16838071 10.1007/s10461-006-9104-8

[23] Kang M Dunbar M Laver S Maternal versus paternal orphans and HIV/STI risk among adolescent girls in Zimbabwe. AIDS Care 2008;20:214–7 18293132 10.1080/09540120701534715

[24] Case A Ardington C The impact of parental death on school outcomes: longitudinal evidence from South Africa. Demography 2006;43:401–20 17051820 10.1353/dem.2006.0022

[25] Timaeus IA Boler T Father figures: the progress at school of orphans in South Africa. AIDS 2007;21:S83–93 18040169 10.1097/01.aids.0000300539.35720.a0

[26] Babalola S Tambashe BO Vondrasek C Parental factors and sexual risk-taking among young people in Cote d’Ivoire. Afr J Reprod Health 2005;9:49–65 16104655

[27] Mott FL Fondell MM Hu PN The determinants of first sex by age 14 in a high-risk adolescent population. Fam Plann Perspect 1996;28:13–8 8822410

[28] Hill C Hosegood V Newell M-L Children’s care and living arrangements in a high HIV prevalence area in rural South Africa. Vulnerable Children and Youth Studies 2008;3:65–77

[29] Hosegood V, McGrath N, Moultrie TA (2009). Dispensing with marriage: marital trends in rural KwaZulu-Natal, South Africa 2000-2006.. Demogr Res.

[30] Halpern-Felsher BL Millstein SG Ellen JM Relationship of alcohol use and risky sexual behavior: a review and analysis of findings. J Adolesc Health 1996;19:331–6 8934293 10.1016/S1054-139X(96)00024-9

[31] Santelli JS Robin L Brener ND Timing of alcohol and other drug use and sexual risk behaviors among unmarried adolescents and young adults. Fam Plann Perspect 2001;33:200–5 11589540

[32] Wellings K Collumbien M Slaymaker E Sexual behaviour in context: a global perspective. Lancet 2006;368:1706–28 17098090 10.1016/S0140-6736(06)69479-8

[33] Wringe A Cremin I McGrath N Comparative assessment of the quality of age-at-event reporting in three HIV cohort studies in sub-Saharan Africa. Sex Transm Infect 2009;**00**(Suppl X):00–0 10.1136/sti.2008.033423 PMC2654104 19307342

[34] Pettifor AE van der SA Dunbar MS Early age of first sex: a risk factor for HIV infection among women in Zimbabwe. AIDS 2004;18:1435–42 15199320 10.1097/01.aids.0000131338.61042.b8

[35] Drain PK Smith JS Hughes JP Correlates of national HIV seroprevalence: an ecologic analysis of 122 developing countries. J Acquir Immune Defic Syndr 2004;35:407–20 15097158 10.1097/00126334-200404010-00011

[36] Kirby D Obasi A Laris BA The effectiveness of sex education and HIV education interventions in schools in developing countries. Ross D Dick B Ferguson J, ed. Preventing HIV/AIDS in young people: a systematic review of the evidence from developing countries Geneva: World Health Organization, 2006:103–50 16921919

[37] Ross D Dick B Ferguson J Preventing HIV/AIDS in young people: a systematic review of the evidence from developing countries WHO Technical Report Series No 938. Geneva: World Health Organization, 2006 16921915

[38] Bearinger LH Sieving RE Ferguson J Global perspectives on the sexual and reproductive health of adolescents: patterns, prevention, and potential. Lancet 2007;369:1220–31 17416266 10.1016/S0140-6736(07)60367-5

